# The genetics of gene expression in complex mouse crosses as a tool to study the molecular underpinnings of behavior traits

**DOI:** 10.1007/s00335-013-9495-6

**Published:** 2013-12-31

**Authors:** Robert Hitzemann, Daniel Bottomly, Ovidiu Iancu, Kari Buck, Beth Wilmot, Michael Mooney, Robert Searles, Christina Zheng, John Belknap, John Crabbe, Shannon McWeeney

**Affiliations:** 1 Portland Alcohol Research Center, Veterans Affairs Medical Center, Portland, 97239 OR USA; 2 Department of Behavioral Neuroscience, Oregon Health & Science University, Portland, 97239-3098 OR USA; 3 Oregon Clinical and Translational Research Institute, Oregon Health & Science University, Portland, 97239-3098 OR USA; 4 Dvision of Bioinformatics and Computational Biology, Medical Informatics and Clinical Epidemiology, Oregon Health & Science University, Portland, 97239-3098 OR USA; 5 Integrated Genomics Laboratory, Oregon Health & Science University, Portland, 97239-3098 OR USA; 6 Division of Biostatistics, Public Health & Preventative Medicine, Oregon Health & Science University, Portland, 97239-3098 OR USA

## Abstract

Complex *Mus musculus* crosses provide increased resolution to examine the relationships between gene expression and behavior. While the advantages are clear, there are numerous analytical and technological concerns that arise from the increased genetic complexity that must be considered. Each of these issues is discussed, providing an initial framework for complex cross study design and planning.

## Introduction

Sandberg et al. (2000) using Affymetrix microarrays, were the first to detect differences in genome-wide brain gene expression between two inbred mouse strains (C57BL/6J [B6] and 129SvEv [129; now 129S6/SvEvTac]). Importantly, these authors observed that some differentially expressed (DE) genes were found in chromosomal regions with known behavioral quantitative trait loci (bQTLs). For example, *Kcnj9* which encodes for GIRK3, an inwardly rectifying potassium channel, was differentially expressed (higher expression in the 129 strain) and is located on distal chromosome 1 in a region where QTLs had been identified for locomotor activity, alcohol and pentobarbital withdrawal, open-field emotionality, and certain aspects of fear-conditioned behavior. This study was unable to address the question of whether or not the elements regulating *Kcnj9* expression were located within the QTL intervals and/or near the gene locus. However, it is possible to extract such causal relationships by combining gene expression and genotype data in genetically segregating populations. Jansen and Nap (2001) were among the first to suggest this approach, which they termed “genetical genomics”. Although originally described for *Arabidopsis*, the strategy was quickly used to examine gene expression in *Drosophila*, yeast, and the mouse (see Lum et al. 2006 and references therein). Schadt et al. (2003) and others defined the expression QTLs (eQTLs) as either *cis* (mapping near the gene locus) or *trans* (mapping elsewhere in the genome). When behavioral QTLs (bQTLs) and *cis*-eQTLs overlap, the *cis*-eQTL genes are inferred as strong quantitative trait gene (QTG) candidates (see e.g. Farris et al. 2010). The situation for *trans*-eQTLs is more complicated since the QTL confidence interval is generally larger and any gene within the QTL interval could have a regulatory role.

The application of genetical genomics to mouse has generally focused on segregating populations involving two inbred strains, one of which is very frequently the B6 strain. Descriptions of these applications are found in the following section. The data analysis is relatively straightforward, especially because good sequence data are available for essentially all strains that would ever be used in a behavioral experiment (Keane et al. 2011). There are, however, problems with the two strain intercross approach. First, two strains will capture only a fraction of the genetic diversity that is available in *Mus* *musculus* (Roberts et al. 2007; Keane et al. 2011). Behavioral techniques and apparatus have been engineered for the placid and some would argue somnambulant laboratory strains of mice that are highly related (Roberts et al. 2007). Using SNPs as a surrogate for genetic diversity, a B6 x DBA/2J (D2) F2 intercross has only 1/6 the gene diversity of a heterogeneous stock (HS) formed from the eight inbred strains used to form the collaborative cross (CC) (Churchill et al. 2004; Iancu et al. 2010); the CC strains include three wild-derived strains. Crosses of low genetic diversity are not optimal for systems biology applications (Churchill et al. 2004; Threadgill and Churchill 2012). Second, given high quality sequence data and dense genotyping platforms, the use of complex crosses allows one to extract for any QTL a haplotype structure which in turn can markedly reduce the QTL confidence interval, in some cases to less than 1 Mbp. Although QTLs of this size are still 1–2 orders of magnitude larger than QTLs detected in human association studies, the reduction in size, especially in gene poor regions, is still sufficient to focus the analysis on a handful of candidates.

This article focuses on the use of complex crosses to examine the relationships between gene expression and behavior. Some historical background is provided as the field has moved from simple to complex segregating populations. While the advantages of complex crosses are obvious, there are several disadvantages, especially ones associated with data analysis. Microarray platforms were not designed for complex crosses and thus, RNA-Seq becomes the preferred strategy for assessing gene expression. While RNA-Seq allows one to examine not only gene expression but also the expression of non-coding RNAs, alternative splicing and allele specific expression, the data analysis is computationally intensive. An additional consideration is that the inclusion of wild-derived strains in the HS-CC has sometimes limited the application of this population for mapping certain behavioral responses. Behavioral testing protocols in mice have been primarily established for assessment in the common laboratory strains and increased locomotor activity associated with the inclusion of the wild-derived alleles has raised concerns about testing validity (see Logan et al. (2013) for recent examination of potential impact in the Diversity Outbred).

## Model systems for complex populations

One could begin a discussion of brain gene expression, behavior, and complex crosses with Sandberg et al. (2000) (see above) but to fully understand the role of mouse complex crosses in this equation, it is perhaps best to start with a series of papers that appeared more than 20 years ago and demonstrated that it was possible to map QTLs for behavioral traits in recombinant inbred (RI) strains of mice (e.g. Gora-Maslak et al. 1991; Belknap 1992). While several RI panels were available, it was the BXD RI panel (Taylor 1978) that was most widely used. These papers and confirmatory F2 intercross studies clearly established two important and related points. One was that the QTL effect sizes were generally small and two, as a consequence, the QTL confidence intervals were typically very large, frequently more than 25 cM (or ~50 Mbp). As a result, it was almost impossible to know which gene or genes within the QTL interval are causally related to the phenotype of interest. This search was of course further complicated at the time by the poor annotation of the mouse genome. Several strategies were developed to reduce the QTL interval (see e.g. Darvasi 1998). These included the use of interval specific congenic strains, mapping in advanced intercross populations, recombinant progeny testing, and the recombinant inbred segregation test. (Talbot et al. 1999, used a variant of the advanced intercross strategy to map QTLs for open-field behavior in a heterogeneous stock (HS) created from eight inbred laboratory mouse strains. A subsequent analysis of these data (Mott et al. 2000) revealed that it would be possible to map QTLs with good precision and extract an approximate QTL haplotype structure. However, despite these and other improvements, only a very small number of behavioral quantitative trait genes (bQTG) have been identified (see e.g. Shirley et al. 2004). Although QTL resolution at the gene level is not typical in some mouse populations, it can be possible to approach gene level resolution in some commercially available outbred populations (Yalcin and Flint 2012) and interval specific congenic lines (Shirley et al. 2004).

Several approaches have been used to identify and prioritize candidate genes within a QTL interval. This initially focused on allelic sequence variation, but, even just a decade ago this was possible only if one was willing to sequence individual genes. Today, given the availability of high quality inbred strain sequence data (Keane et al. 2011), it is now possible to interrogate a QTL interval and determine which genes harbor non-synonymous coding SNPs that match the QTL profile. An alternative approach, which was widely adopted, was to integrate QTL analysis and gene expression profiling, emphasizing the genetical genomics approach (Jansen and Nap 2001). The emphasis on this approach was key to the development of WebQTL (Wang et al. 2003). Gene expression data from multiple brain regions was made available for the B6 and D2 inbred strains and 32 BXD RI strains. Also posted at the Web site were a variety of RI strain behavioral and genotype data. For many investigators, this was the first portal for examining how the natural variation in gene expression and behavior were correlated. Over the years, the Website has been updated by the inclusion of brain gene expression data from other RI panels, mouse F2 intercrosses, additional BXD RI strains, and a significant number of inbred mouse strains, including whole brain and brain regional data. The data have been used in a variety of ways, including detecting how patterns of gene co-expression have behavioral associations (Chesler et al. 2005).

Peirce et al. (2006) mined the data to address the question of “how reliable are eQTLs?”. These authors noted that for B6xD2 genotypes, *cis*-eQTLs are highly replicable but that there is an overabundance on eQTLs where the B6 strain is associated with higher expression. These data suggested that some of these QTLs were artifacts due to SNPs and the poor hybridization of the D2 cDNA. Subsequent experiments showed that indeed this was the case (Walter et al. 2007, 2009). Flint and colleagues (see Solberg et al. 2006; Valdar et al. 2006a, b) mapped QTLs for a variety of behavioral phenotypes in >2,000 HS animals; this HS population (HS/NPT), also an eight strain cross, differed from that used by Talbot et al. (1999). Importantly for this article, Flint and colleagues collected hippocampal gene expression data on 460 animals (Huang et al. 2009). Similar to Peirce et al. (2006), Huang et al. (2009) concluded that a significant proportion of the *cis*-eQTLs were hybridization artifacts. Nonetheless and not unexpectedly, the number of “true” *cis*-eQTLs appeared to be significantly greater than those previously detected in simpler crosses; i.e., in the HS population, additional regulatory alleles are detected. Similar results were obtained for gene expression in a simpler HS (HS4), derived from crossing four laboratory strains (Malmanger et al. 2006).

The CC (Churchill et al. 2004) was formed to provide a unique system biology resource that addresses many shortcoming in available mouse strain resources, such as limited genetic diversity. The goal was to generate >1,000 RI strains formed from eight inbred strain founders that capture >90 % of the genetic diversity available in *Mus musculus*. Three of the CC founders are wild-derived strains. Although it appears that only several hundred RI strains will reach completion, the CC, like the BXD RI panel, will in time provide an important reference population for examining gene-behavior relationships. Two outbred versions of the CC have been created, the HS-CC and the Diversity Outbred (DO) (Iancu et al. 2010; Churchill et al. 2012). To date, brain gene expression data are only available for the HS-CC. Iancu et al. (2010) compared brain (striatum) gene expression in a B6xD2 F2 intercross, HS4, and HS-CC animals. Although it was assumed that the regulation of gene expression would differ in each of the populations, it was also assumed that given striatal function is not cross dependent, at some level function and gene expression should overlap in a similar way for all three crosses. To address this issue, Iancu et al. (2010) utilized the Weighted Gene Co-expression Network Analysis (WGCNA) (Zhang and Horvath 2005). This analysis builds from the premise that (a) gene expression networks have scale free properties (i.e. there are a few highly connected nodes) and (b) co-expressed genes share similar functions. The analysis revealed that while there were some cross-dependent differences, the overall modular substructure of the co-expression network was cross independent, the highly connected nodes remained intact. Iancu et al. (2013) next asked if selection for a behavioral phenotype (haloperidol-induced catalepsy) had similar effects on expression network structure across the three crosses. The results obtained are both interesting and cautionary as we press forward examining complex cross gene expression. The selection paradigm was short-term (3–4 generations), the rate of segregation of the responsive and non-responsive lines was similar, and the responsive and non-responsive lines all differed by greater than 20-fold in the haloperidol ED50. The difference in response was not pharmacokinetic. The first key observation was that there was no overlap of differential gene expression for the three selections. The second key observation was that as genetic diversity increased, the number of co-expression modules affected by selection also increased. It was possible to identify a core set of modules affected by selection. What is unknown is whether or not the additional modules that were affected by selection e.g. in the HS-CC population, are relevant to our understanding of the gene-behavior relationship.

## Phenotype measurements in eQTL analysis

Several technological advances have fundamentally altered the definition of phenotype in QTL studies. Mapping RNA transcript and protein abundance levels is widespread, and in principle any biologic characteristic of interest can be tested for association with genetic polymorphisms. In the context of neurobehavioral traits, examples include number of neuronal cells in specific brain regions (Rosen and Williams 2001; Airey et al. 2001) and also brain morphometry (Li et al. 2005; Jan et al. 2008). The focus of this review is on high-throughput methodologies and in particular measurements of gene expression such as microarrays, qPCR, and RNA-Seq. While these technologies offer tremendous breath to transcriptome analysis, several factors can adversely affect the quality of the results. All technologies assume intact RNA; the extent to which this assumption is true can be evaluated using the RNA integrity number (RIN) (Schroeder et al. 2006). From human studies, it has been shown that possible confounding factors include length of time post-mortem and the pH of the sample; statistical analysis can incorporate these as covariates (Liu 2011). For hybridization based methods, factors affecting probe matching can strongly affect expression measurement (Walter et al. 2007); these errors can further propagate in the course of eQTL mapping (Iancu et al. 2012). PCR based methods can also be affected by polymorphisms within the primer sequence. Taking into account these factors has beneficial effects on the downstream analysis.

Batch effects can introduce serious confounding factors in the analysis of expression levels; ideally, all samples should be processed at the same time. If separate batches are unavoidable, balancing case/controls, and sex within batches is important. Several techniques that alleviate batch effects have been proposed, with the ComBat package among the most popular (Johnson et al. 2007).

A major limitation affecting microarray-based analyses is the limited dynamic range of the fluorescence signals. This problem is resolved by the RNA-Seq methodology, where the dynamic range is orders of magnitude above the microarray capacity (Nagalakshmi et al. 2008). The adverse effects of SNPs on probe hybridization are also completely alleviated by RNA-Seq. Count data is directly related to expression level, as opposed to microarrays where the fluorescent intensity is an indirect measurement. Although RNASeq is more costly than array-based technologies, costs are steadily decreasing, which promises increased utilization of this technology.

## Analytical approaches for eQTL

The analysis of eQTL in complex crosses mirrors that of traditional QTL mapping at its core. However, it also comes with additional issues that require special care by an analyst either not considered in the simplest forms of QTL mapping or further exacerbated. We will briefly review some of the most common choices of statistical methodology with an emphasis on methods for the analysis of crosses with more than two founders. First, we will consider common issues between high dimensional eQTL techniques. Specifically we will consider methods devised to deal with the multitude of statistical tests that need to be performed for a given experiment through either corrections to significance measures or by approaches that reduce the number of tests that need to be performed. We will then discuss specific statistical methodology devised for the analysis of the emerging RNA-Seq technology as related to more established microarray eQTL methods. Note that this review will mainly consider frequentist methods, though we note that Bayesian approaches are becoming more prevalent in mouse genetics. See, for instance, the review by Stephens and Balding (Stephens and Balding 2009) as an introduction to Bayesian methods in genetics. Also note that we focus on the case of a single QTL/eQTL underlying a given trait though generalizations of the below methodology allow the examination of two or more loci.

### Overview of genetic and statistical considerations

**Fig. 1:**
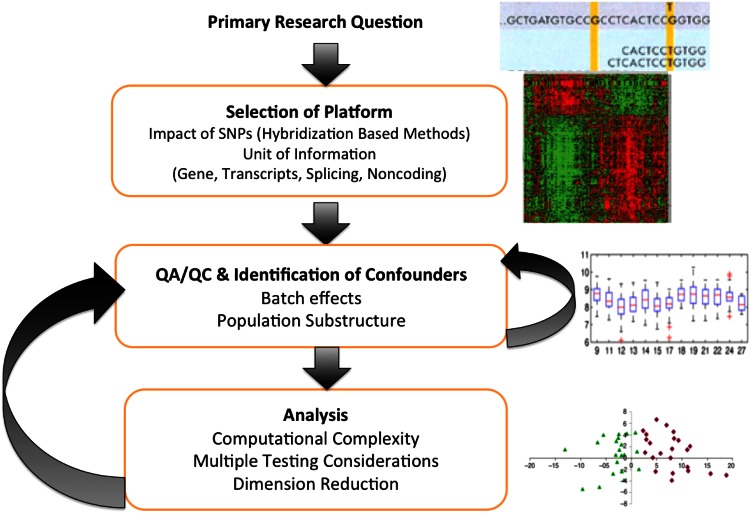
Simple framework that highlights (in each *orange box*) the design and analysis considerations that should be taken into account for expression studies utilizing complex crosses. It is noted that the primary research question, as well as the cross, accompanying assumed genetic model and phenotype must be determined first

The analytical methods with which QTL/eQTL analysis occurs depends on the cross as well as other experimental factors such as the assumed genetic model and phenotype. It is important to note that there are a number of design considerations that should be taken into account early in the planning process, particularly for studies utilizing complex crosses (Fig. 1). For crosses involving two inbred progenitor mouse lines (i.e. F2s intercross or backcrosses) either a single marker analysis of variance (Broman and Speed 1999), interval mapping (Lander and Botstein 1989), or related regression based approaches (Haley and Knott 1992) are typically applied when assuming the presence of a single QTL. For crosses with more than two inbred founders such as in heterogenous stock (HS) (McClearn et al. 1970), CC (Churchill et al. 2004) or Diversity Outbred (DO) (Svenson et al. 2012) mice, typically multiple regression procedures are performed based on estimates of founder strain allelic contributions for a given marker/interval (Talbot et al. 1999; Mott et al. 2000; Svenson et al. 2012; Aylor et al. 2011; Durrant et al. 2011; Philip et al. 2011). These values are the result of haplotype reconstruction in terms of the founder lines using either the genotype calls (Mott et al. 2000; Liu et al. 2010), or intensities of the genotyping arrays (Svenson et al. 2012; Collaborative Cross 2012). Haplotype reconstructions in this manner mainly draw on the use of a Hidden Markov Model though alternate approaches have also been recently considered (Zhou et al. 2012). Hidden Markov Models are a machine learning approach designed for inferring underlying states of an unknown spatially/temporally ordered variable (Rabiner 1989). For this application, the states would correspond to founder inbred strain haplotypes and the end result would be a matrix of probabilities of descent from each pair of founder inbred strains which can be further summarized per strain (Mott et al. 2000; Valdar et al. 2009). The basic multiple linear regression model approach in this case would typically compare a model with the founder contributions to one without the founder contributions for each marker interval. The comparison of these two models allow the computation of an F statistic and accompanying *p* value (Valdar et al. 2009).

### Multiple testing considerations

One issue that is exacerbated in high dimensional eQTL scans is how to pick a significance threshold once *p* values (or LOD scores) are generated for each expression phenotype. The way in which these thresholds are chosen can be roughly divided into three categories ordered by decreasing conservativeness: familywise error rate, false discovery rate (FDR), and permutation/simulation procedures. The procedure used depends on the expected effect size as well as type of desired downstream analysis. For instance if the main goal is to confirm the top ranked genes via qPCR there is little benefit to incur the increased computational and analytical time generating and interpreting large lists of genes potentially regulated by an QTL. Therefore a familywise based approach such as the Bonferroni correction would make sense (Bottomly et al. 2012). The Bonferroni correction has also been used as an approach to estimate the number of false positives (Schadt et al. 2003).

Controlling the false discovery rate also has been suggested (Storey and Tibshirani 2003; Carlborg et al. 2005). A common way to implement this control is through the computation of *q* values from the scan *p* values. A *q* value corresponds to the expected proportion of false positives when calling a given test significant (Storey and Tibshirani 2003). It has been used on top of permutation-based *p* values as a way to estimate the specificity of the given scan (Aylor et al. 2011; Chesler et al. 2005). In addition, FDR values have been estimated directly using subsets of the eQTL *p* values (Ghazalpour et al. 2008). One issue with considering FDR corrections is the presence of dependence if multiple *p* values are considered per expression trait (Kendziorski and Wang 2006). Dependence between two tests in this context means that say, a low *p* value for trait A implies a low *p* value for trait B. For instance the computation of *q* values relies on at most weak dependence between *p* values and violations of this may cause inaccuracies of the method (Storey and Tibshirani 2003). However, application of an approach such as surrogate variable analysis could be applied to remove dependencies between the test statistics increasing the validity of the *q* values (Leek and Storey 2007, 2008).

Permutation testing is arguably the most common approach for significant assessment in eQTL studies. An approach similar to QTL studies would apply a permutation procedure to each expression trait separately (Churchill and Doerge 1994). However, as the number of tests is thousands of times greater than a standard QTL analysis, it is not desirable to perform a full permutation test potentially increasing computation time by at least an additional thousand-fold. One approach is to reduce the number of permutations necessary to compute the significance threshold through the use of a parametric model (Valdar et al. 2006a). Also, permutation testing procedures can be applied to only a subset of expression traits with the result then used to choose thresholds for the remaining traits (Huang et al. 2009; Aylor et al. 2011). This approach needs to take into consideration distributional differences among the traits that can lead to large differences in threshold values (Carlborg et al. 2005). One approach to choose representative threshold values is to interpolate based on a representative group of threshold values (Huang et al. 2009), another is to choose a global threshold based on the distribution of the thresholds (West et al. 2007). Regardless of the approach used to generate the significance thresholds, permutations need to be carried appropriately out with regard to experimental design (Churchill and Doerge 2008).

### Dimension reduction

One strategy to reduce the number of tests being performed in an eQTL setting is to focus only on a subset of expression traits relevant to the phenotype(s) of interest. Relevance in this case is determined through differential expression analysis (Schadt et al. 2003). Other approaches take advantage of the fact that expression data is highly correlated to first form groups of genes with highly similar expression profiles followed by a QTL mapping procedure, two common procedures for doing this are clustering and principle component analysis. Clustering algorithms are commonly used in microarray experiments (Eisen et al. 1998) and have been used successfully as a means to reduce the number of traits necessary to map (Chun and Keleş 2009; Lan et al. 2003; Yvert et al. 2003). Procedures based on principal components analysis, which seeks to find eigengenes or eigentraits that explains a certain amount of variability while being independent from one another (Alter et al. 2000), have also been applied to expression data prior to mapping (Lan et al. 2003; Biswas et al. 2008). Mapping expression traits by first clustering the expression data and then summarizing the clusters using the ‘eigengene’ have also been shown to be effective for finding QTL regions with a large effect on expression traits (Fuller et al. 2007).

### RNA-Seq eQTL approaches

The advent of microarrays made eQTL approaches an attractive option to elucidate the genetic underpinnings of gene expression. However, microarrays have many issues that prevent them from being an ideal datasource. For instance, microarrays have fixed probes/reporters that can limit expression estimates. This means both that a potential gene of interest may not be interrogated in addition to the possibility that hybridization of the probes on the array may be affected by genomic differences as is discussed later. A more recent approach is the high throughput sequencing of the mRNA population in a given experimental condition for a given animal (Mortazavi et al. 2008). This data source is less constrained by annotation, is free from relying on reporter hybridization and therefore allows additional types of analyses related to basic microarray-based eQTL to be performed.

The first type of analysis facilitated by RNA-Seq is the study of transcript-level expression specifically alternative splicing QTL (sQTL) as has been found to be informative in humans (Heinzen et al. 2008; Kwan et al. 2008). This type of analysis has been examined using microarrays for complex mouse crosses (Alberts et al. 2005), however, in practice fixed microarray probe placement and genomic differences between probe sequence and RNA source was a major impediment (Huang et al. 2009; Ciobanu et al. 2010). From recent studies using RNA-Seq, it appears that the technology is better suited to assessing the genetics of alternative splicing analysis in humans (Pickrell et al. 2010; Rakitsch et al. 2012). However though it has been suggested as a promising avenue of research (Guryev and Cuppen 2009; Hitzemann et al. 2013) little work appears to have been done applying the method to mouse crosses.

Another potential benefit to the use of RNA-Seq is the direct study of allele-specific expression. These experiments have traditionally been performed through the use of RT-PCR based confirmation approaches (Cowles et al. 2002). Allele-specific expression is implemented in practice for RNA-Seq in a similar manner by essentially counting the number of sequence reads generated by the technology that overlap with either the reference or alternative allele(s) (Degner et al. 2009). Initial applications of this approach to study embryonic imprinting yielded promising (Gregg et al. 2010) though conflicting messages (DeVeale et al. 2012) about the additional power RNA-Seq lends to the problem.

### Computational issues

One of the central issues with eQTL mapping is the drastic increase in computational capabilities it requires over a similar QTL study. This is only exacerbated by increases in marker density of new genotyping arrays (Yang et al. 2009) and expression traits in exon-level oligonucleotide arrays (Gardina et al. 2006) or RNA-Seq (Mortazavi et al. 2008). In order to gain computational efficiency, aspects of the underlying mathematics can be leveraged to provide essentially the same results using less computational resources. The simplest example of this is the ability to use a matrix of phenotypes in standard linear model fitting as opposed to a single phenotype vector as is typically used. This means that relatively computationally expensive matrix calculations are performed only once and can therefore be leveraged to perform batch processing of phenotypes at a significant decrease in computational time (Valdar et al. 2009). This type of batch processing also lends itself to parallel processing either through a cluster computing environment or a single computer with multiple processors. A related example is the mixed effects model framework of EMMA (Kang et al. 2008). Similarly, analysis methods have also been developed for RNA-Seq that make computationally beneficial approximations to the underlying parameter estimation procedure (McCarthy et al. 2012).

### Population substructure

Population substructure is a serious confounding factor in many QTL and eQTL mapping studies (Devlin et al. 2001; Pritchard and Donnelly 2001; Kang et al. 2008; Valdar et al. 2009; Listgarten et al. 2010). In brief, the problem can be summarized as follows: for a statistical test used to identify the causative genetic effects on a phenotype, the null hypothesis states that there is no association between the genetic locus and the phenotype. However, this assumption does not hold in cases where population substructure is present: differences in average phenotype value between the subpopulations will be detected as a QTL for each genetic locus that segregates between the subpopulations, even though the locus is not necessarily causative. It is therefore important to distinguish between causative associations and associations due solely to genetic linkage.

In mouse QTL studies, much of the uneven relatedness between individuals is due to the complex genetic history of the commonly used inbred strains. The most significant differences are between the classical inbred strains and the wild-derived inbred strains (Ideraabdullah et al. 2004; Yalcin et al. 2004). Classical inbred strains are derived from a limited number of individuals of the *Mus musculus* subspecies that have widely varying degrees of relatedness (Bonhomme et al. 1989). The wild-derived strains are derived from several Mus subspecies captured at different times and geographic locations (Bonhomme and Guenet 1989). Therefore, studies that evaluate phenotypic variability among several inbred strains need to account for the phylogenetic differences.

Heterogeneous stock mice are derived from inbred strains using various outbreeding procedures (Chia et al. 2005). QTL mapping in these populations offers markedly higher resolution as compared to simple intercrosses (Talbot et al. 1999; Svenson et al. 2012). However, despite efforts to randomize the mating process, individuals in outbred mouse populations display varying levels of relatedness (Aldinger et al. 2009; Iancu et al. 2012). Furthermore, an in-depth analysis of the structure of a heterogeneous stock mouse population revealed that relatedness is not evenly distributed across the genome and individual chromosomes can have effects on phenotype that are distinct from the whole genome kinship information (Iancu et al. 2012) adding another layer of complexity. Therefore, mapping strategies employed in outbred populations need to adjust for this confounding factor (e.g., Cheng et al. 2011 and references therein).

Attempts to adjust for population substructure fall into several categories. In human association studies, genomic control (Devlin et al. 2000) structured association (Pritchard et al. 2000) and principal component analysis (Patterson et al. 2006) are the most commonly employed procedures. In mouse populations, the relatively large effect size of the kinship structure seems to favor an alternative mixed-model approach (Kang et al. 2008). In a further refinement of this approach (Iancu et al. 2012), we recently demonstrated that it is possible to simultaneously detect strain-specific effects and also correct for population structure.

### Causal inference

One of the main benefits of eQTL studies is the ability to form networks based on the correlation/covariation structure of the expression data across the experimental populations (Chesler et al. 2005). This allows relationships between expression traits to be expressed, for example, Trait A and Trait B are correlated and therefore there is potentially a relationship between the two traits. Without additional information or assumptions typically one cannot state confidently whether Trait A causes Trait B (Trait A→Trait B) or Trait A reacts to Trait B (Trait A←Trait B) or whether there is a confounding factor responsible for the observed correlation. Therefore co-expression networks by themselves cannot usually be used to form ‘causal’ or ‘reactive’ hypotheses, however when jointly considered with DNA variation data such inference is possible (Schadt et al. 2005). The inclusion of DNA variation data in the context of experimental crosses is necessary as it can be assumed to be the main driver of variation in the traits under consideration (Schadt et al. 2005). There are several similar ways in which causal reasoning is performed in the eQTL context: model selection approaches (Schadt et al. 2005; Chen et al. 2007; Millstein et al. 2009) structural equation modeling (SEM) (Liu et al. 2008; Aten et al. 2008) and Bayesian networks (Zhu et al. 2007). All of these approaches are similar in spirit in that they attempt to define local or global relationships of the form Marker A→Trait B→Trait C. Although, the use of causal inference approaches have shown promise, in general some cautions apply about the interpretation of causal modeling in eQTL. Specifically, consideration of large sample sizes, the removal of factors that can play a role as a hidden confounder as well as considering comprehensive sets of models are seen as necessary steps for robust causal modeling (Li et al. 2010).

## Conclusion

The utility and value of complex crosses for examining the relationship between behavior and expression is clear. However, there are numerous considerations given the increased genetic complexity that must be dealt with in the design of these types of studies. By highlighting each of these, we provide a conceptual framework to guide researchers in study planning and implementation.
